# Pathophysiology, Diagnosis, and Ablation of Atrioventricular Node–dependent Long-R–P Tachycardias

**DOI:** 10.19102/icrm.2020.110306

**Published:** 2020-03-15

**Authors:** Max Weiss, Reginald T. Ho

**Affiliations:** ^1^Department of Medicine, Division of Cardiology, Thomas Jefferson University Hospital, Philadelphia, PA, USA

**Keywords:** atrioventricular nodal reentrant tachycardia, long-R–P tachycardia, nodofascicular, nodoventricular, permanent form of junctional reciprocating tachycardia

## Abstract

Atrioventricular (AV) node–dependent long-R–P tachycardias are a unique group of supraventricular tachycardias that include atypical AV nodal reentrant tachycardia (AVNRT), atypical AVNRT with a concealed bystander nodofascicular (NF)/nodoventricular (NV) accessory pathway inserting into the slow pathway of the AV node, the permanent form of junctional reciprocating tachycardia, and orthodromic NF/NV reciprocating tachycardia. Here, we discuss the complex pathophysiology, diagnosis, and ablation of these intriguing arrhythmias.

## Introduction

**Figure 1:**
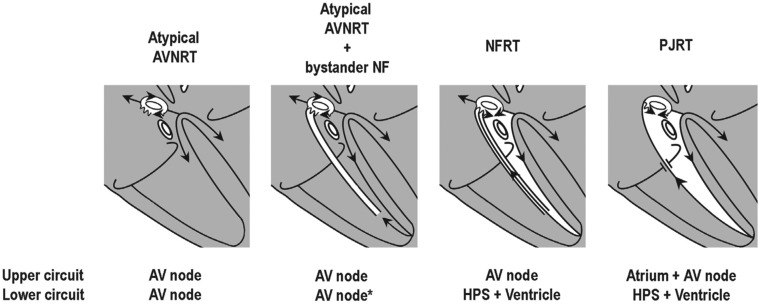
Diagram illustrating the four AV node-dependent long-R–P tachycardia circuits. *NF/NV AP–SP allows a His-refractory VPD to reset or terminate an atypical AVNRT.

Atrioventricular (AV) node–dependent long-R–P tachycardias are a unique and complex group of supraventricular tachycardias that include atypical AV nodal reentrant tachycardia (AVNRT), atypical AVNRT with a concealed bystander nodofascicular (NF)/nodoventricular (NV) accessory pathway inserting into the slow pathway (SP) of the AV node, the permanent form of junctional reciprocating tachycardia (PJRT), and orthodromic NF/ventricular reciprocating tachycardia (NFRT/NVRT) **(Figure 1)**.^1^

## Pathophysiology

In the context of these unusual tachycardias, the R–P intervals are long (R–P > P–R) because retrograde conduction occurs over the SP, a slowly conducting AV accessory pathway (AP), or via an AP inserting into the SP (NF/NV AP–SP). As mentioned, four known tachycardia mechanisms include atypical AVNRT, atypical AVNRT with a bystander concealed NF/NV AP–SP, PJRT, and NFRT/NVRT.

### Atypical atrioventricular node reentrant tachycardia

Classic atypical (fast–slow) AVNRT is the most common AV node–dependent long-R–P tachycardia. It utilizes the fast pathway (FP) and SP of the AV node as the antegrade and retrograde limbs of the reentrant circuit, respectively, and its nonmacroreentrant circuit is confined to the AV node and its atrionodal inputs. The SP is typically located along the right posteroseptum (right inferior extension), although left-sided and superior SPs have also been described.^2^

### Atypical atrioventricular node reentrant tachycardia with a bystander concealed nodofascicular/nodoventricular accessory pathway inserting into the slow pathway

As another example, a concealed NF/NV AP could insert into the SP of the AV node (NF/NV AP–SP) and serve as a bystander during atypical AVNRT.^3–5^ This bystander pathway allows His-refractory ventricular premature depolarizations (VPDs) to reset or terminate atypical AVNRT, leading to the potential misdiagnosis of an AP-mediated tachycardia (PJRT or NFRT/NVRT).

### Permanent form of junctional reciprocating tachycardia

PJRT is an atypical form of orthodromic reciprocating tachycardia (ORT) that relies on a slowly conducting, decremental AV AP.^6–8^ Because the AP bridges the AV groove, the atrium is an integral part of the circuit. These APs are classically (but not always) located along the posteroseptum, resulting in a midline atrial activation pattern that mimics atypical AVNRT.

### Nodofascicular/ventricular reciprocating tachycardia

NFRT/NVRT is another unusual type of macroreentrant ORT that uses an NF/NV AP, with the nodal pathway inserting into the SP of the AV node.^9^ Unlike with PJRT, however, here, the atrium is not an integral part of the circuit. The distal insertion is generally in (NF) or near (NV) the right bundle (RB).

## Diagnosis

**Figure 2:**
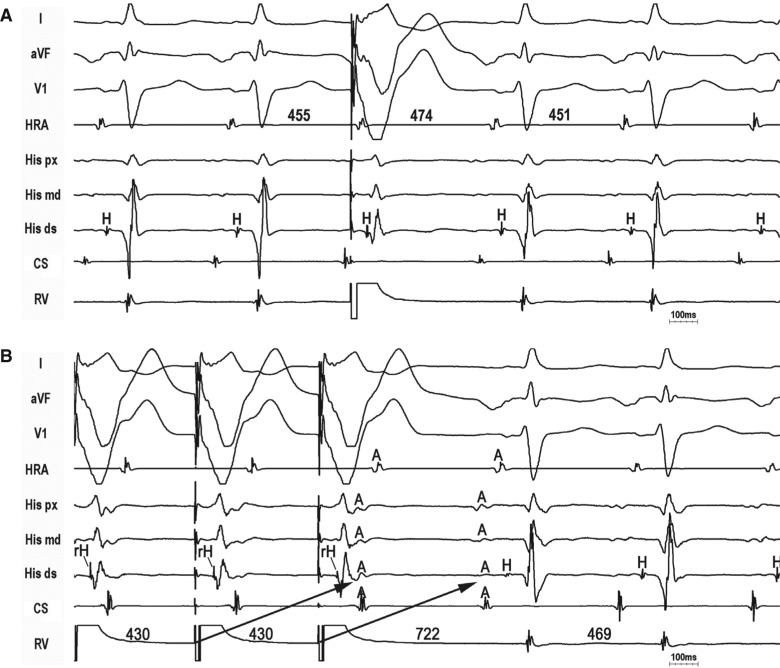
PJRT. **A:** A His-refractory VPD delays the atrium by 19 ms, indicating the presence of a slowly conducting decremental AP. **B:** Entrainment from the ventricle with antidromic capture of the His bundle causes a pseudo-A–A–V response and PPI − TCL = 253, causing potential misdiagnosis of atypical AVNRT.

**Figure 3:**
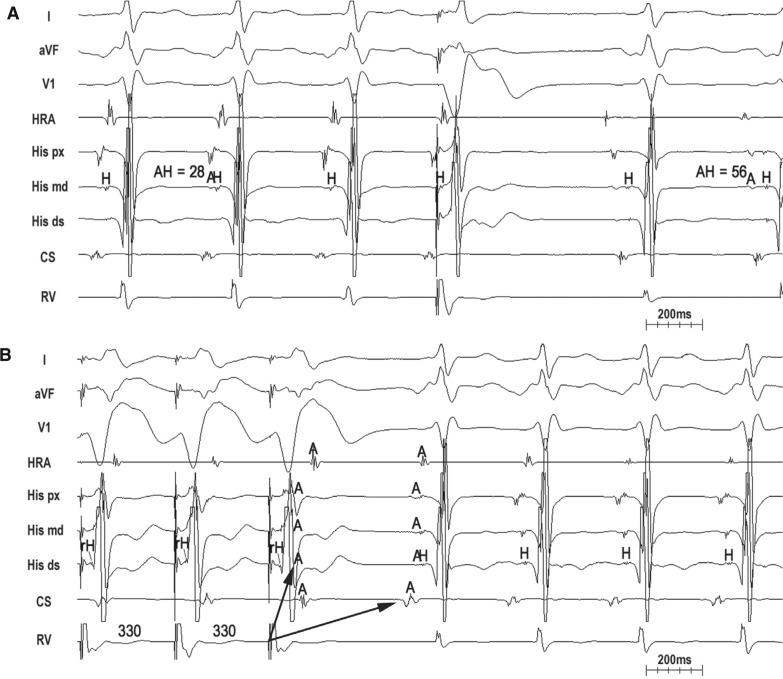
Atypical AVNRT with bystander NF/NV AP–SP. **A:** A His-refractory VPD terminates tachycardia with a VA block, proving the presence of an AP. Paradoxically, AH _(SVT)_ < AH _(NSR)_, excluding PJRT. **B:** Entrainment from the ventricle with a true A–A–V response due to retrograde “double-fire” (FP and NF/NV AP–SP) and long PPI – TCL (> 125 ms). Despite retrograde capture of the His bundle, the atrium is not advanced until the last paced complex, indicating that the His bundle is not part of the circuit and firmly excludes NFRT.

**Figure 4:**
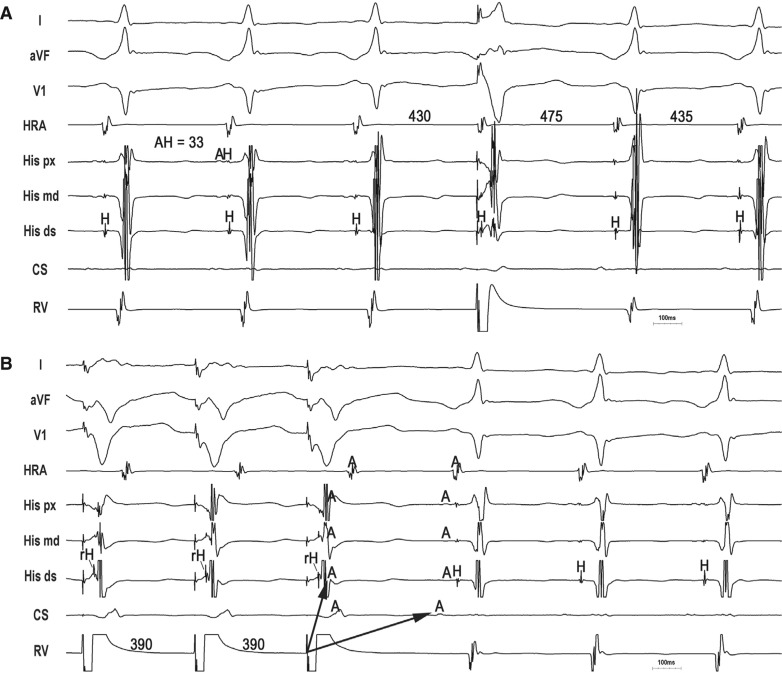
Atypical AVNRT with bystander NF/NV AP–SP. **A:** A His-refractory VPD delays the atrium by 45 ms, indicating the presence of a slowly conducting decremental AP. The very short AH interval (pseudo-interval) excludes PJRT. **B:** Entrainment from the ventricle with a true A–A–V response due to retrograde “double-fire” (FP and NF/NV AP–SP) and long PPI – TCL (> 125 ms).

Slow, decremental conduction over the SP/AP brings into focus unique challenges for the diagnosis of long-R–P tachycardias that are not encountered in relation with short-R–P counterparts. First, spontaneous termination with AV block is uncommon. Though reproducible, spontaneous termination of a short-R–P tachycardia with AV block indicates AV nodal dependency and excludes atrial tachycardia, long-R–P tachycardias more commonly terminate in the retrograde limb (SP/AP), which is less helpful for diagnosis. Second, Coumel’s law may be absent. Although bundle branch block (BBB)-induced prolongation of the VA interval and tachycardia cycle length (TCL) indicates ORT with an AP ipsilateral to BBB,^7,10^ Coumel’s law is less frequently seen with PJRT/NFRT because long-R–P tachycardia rates are slower and less susceptible to acceleration-dependent BBB; the loss of BBB and transeptal conduction time are counterbalanced by slower, decremental AP conduction (and vice versa); and the septal location of these APs minimizes the effect of BBB. Third, A–A–V responses are common. A–A–V responses following the entrainment of long-R–P tachycardia from the ventricle are relatively common and can cause a misdiagnosis of atrial tachycardia.^11^ Pseudo-A–A–V responses occur when pacing-induced decrement over the SP (atypical AVNRT) or slowly conducting AP (PJRT/NFRT) cause paced VA intervals to exceed the pacing cycle length (V–A > V–V) such that the first atrial activation upon pacing cessation results from the penultimate pacing stimulus **(Figure 2)**.^6^ Pseudo-A–A–V responses are true A–V responses and are identifiable by determining the last entrained atrium. True A–A–V responses also occur as a result of retrograde dual conduction over the FP and slowly conducting SP/AP (ie, retrograde “double-fire”) **(Figures 3 and 4)**.^4,5,12^ Lastly, ventricular pacing maneuvers during sinus rhythm (para-Hisian/differential RV pacing) are less helpful than those during tachycardia (His-refractory VPDs/RV entrainment).^13,14^ The reasons for this are as follows: (1) the FP can preempt SP/AP conduction and prevent identification of the slowly conducting structure responsible for tachycardia; (2) 1:1 SP/AP conduction may not occur at the slowest pacing rate allowable by sinus rhythm; and (3) para-Hisian pacing over an NF AP–SP can elicit an “AV nodal” response because retrograde conduction is dependent upon RB capture.^15^ Similarly, differential RV pacing can produce an “AV nodal” response when retrograde conduction occurs over long, insulated AV AP or NF/NV AP–SP inserting closer to the RV apex than base.^16^

### Identifying an accessory pathway

Critical to a long-R–P tachycardia diagnosis is establishing the retrograde limb of the circuit—that is, SP (AVNRT), AV AP (PJRT), or NF/NV AP–SP (NFRT/NVRT). His-refractory VPDs are the maneuver of choice to identify the presence of an AP.^17^ The three positive responses of His-refractory VPDs proving the presence of an AP are: (1) resetting with advancement, (2) resetting with delay, and (3) termination with VA block **(Figures 2–4)**. Resetting with delay is least common, only observed with long-R–P tachycardias, and indicates an AP with significant decremental properties (ie, the degree of VPD prematurity is offset by a greater degree of AP delay).^6,18^ Such a decremental AP can result in (1) pseudo-A–A–V responses following entrainment from the ventricle (causing a potential misdiagnosis of AT) and (2) apparent lack of resetting when the degree of VPD prematurity is offset by an equal degree of AP delay (ie, full compensation, causing a potential misdiagnosis of atypical AVNRT). While any positive His-refractory VPD response proves the presence of an AP, such does not prove AP participation in tachycardia. Atypical (fast–slow) AVNRT can be reset or terminated with VA block by a His-refractory VPD in the presence of a bystander NF/NV AP inserting into the SP.^3–5^

### His-refractory ventricular premature depolarization equivalents

**Figure 5:**
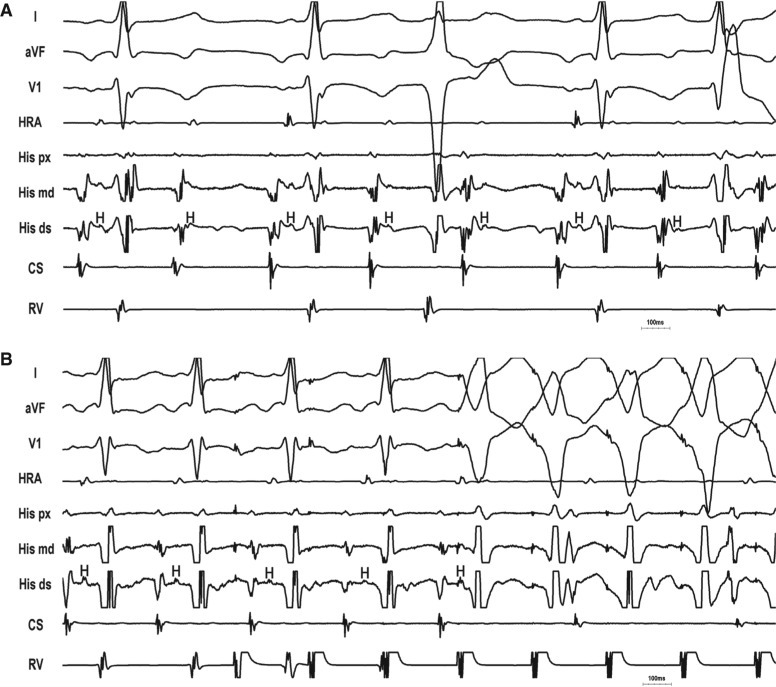
Atypical AVNRT with bystander NF/NV AP–SP. **A:** Persistence of tachycardia despite AV block excludes PJRT/NFRT/NVRT. **B:** With the onset of VOP, the first paced complex (His-refractory) terminates the tachycardia with VA block, indicating the presence of an AP. With pure atypical ORT excluded, the diagnosis is atypical AVNRT with a bystander NF/NV AP inserting into the SP.

**Figure 6:**
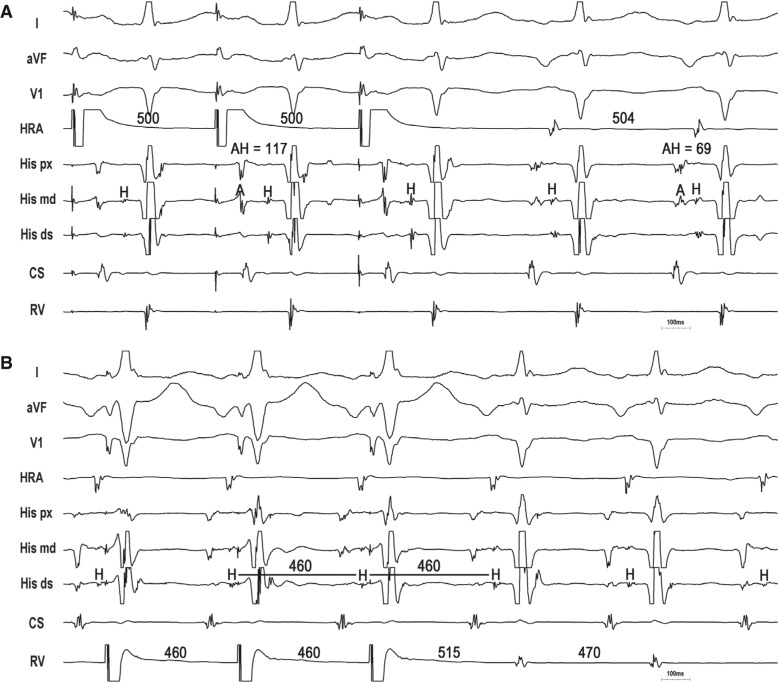
NVRT. **A:** Entrainment from the atrium results in ∆AH = 48 ms, indicating nodal tachycardia. **B:** Entrainment from the ventricle with orthodromic capture of the His bundle and short PPI – TCL = 45 ms indicates a macroreentrant NFRT/NVRT and excludes atypical AVNRT. The presence of paced QRS fusion with relatively narrow-paced QRS complexes favors NVRT over NFRT.

Two other methods to identify the presence of an AP include resetting or termination of the tachycardia within the transition zone (TZ) during the onset of ventricular overdrive pacing (VOP) and entrainment from the ventricle with orthodromic capture of the His bundle **(Figures 5 and 6)**.^1,19^ During the onset of VOP, the TZ represents fusion between paced and tachycardia wavefronts so that all QRS complexes are, by definition, His-refractory. Any TZ complex that perturbs the tachycardia therefore indicates the presence of an AP. Similarly, entrainment of the tachycardia from the ventricle with orthodromic capture of the His bundle also proves the presence of an AP. Orthodromic His capture occurs when the collision point between orthodromic and antidromic wavefronts is below the His bundle and is the equivalent of continuous resetting of tachycardia by repetitive His-refractory VPDs.

### Delineating the entire circuit

Critical to establishing the diagnosis of an AV node-dependent long-R–P tachycardia is establishing the upper and lower limbs of its circuit.^1^ The upper limb is the AV node for nodal tachycardias (atypical AVNRT/NFRT/NVRT), while involvement of the atrium and AV node is required for PJRT **(Figure 1)**. Additionally, the lower limb is the AV node for AVNRT but the His–Purkinje system (HPS) and ventricle for atypical ORT (PJRT/NFRT/NVRT) **(Figure 1)**.

### Upper limb: atrioventricular node (nodal tachycardias) versus atrium + atrioventricular node (permanent junctional reciprocating tachycardia)

PJRT is the only AV node-dependent long-R–P tachycardia where the atrium is an integral part of the circuit. This means that dissociation of the atrium from tachycardia excludes PJRT and atrio–His (AH) intervals are true intervals reflecting sequential activation of the atrium and His bundle over the AV node. Similarly, AH intervals during entrainment/pacing from the atrium at the TCL are true sequential intervals so that ∆AH (AH _(entrain/pace @ TCL)_ − AH _(PJRT)_) < 20 ms.^20^ In contrast, AH intervals for nodal tachycardias (atypical AVNRT/NFRT/NVRT) are pseudo-intervals reflecting simultaneous activation of the atrium (retrogradely) and FP/His bundle (antegradely). Nodal tachycardias, therefore, can have very short AH intervals, which are paradoxically shorter than those seen during sinus rhythm **(Figures 3 and 4)**.^4,5^ Moreover, ∆AH (AH _(entrain/pace @ TCL)_ − AH _(nodal tachycardia)_) > 40 ms.^20^

### Lower limb: atrioventricular node (atypical atrioventricular node reentrant tachycardia) versus His–Purkinje system and ventricle (atypical orthodromic reciprocating tachycardia)

Atypical AVNRT is the only AV node-dependent long-R–P tachycardia confined to the AV node (a nonmacroreentrant circuit not incorporating the HPS/ventricle as an integral part of the circuit). This means that persistence of an AV node-dependent long-R–P tachycardia despite AV block indicates atypical AVNRT and excludes atypical ORT, while postpacing interval (PPI) − TCL < 125 ms identifies macroreentry, thereby excluding atypical AVNRT **(Figures 5 and 6)**. Classically, a PPI − TCL cutoff value of 115 ms has been used to differentiate the short-R–P tachycardias.^21^ However, for long-R–P tachycardias, a PPI − TCL cutoff value of 125 ms seems to better discriminate atypical AVNRT from PJRT/NFRT/NVRT because atypical ORT can generate long PPIs for several reasons, including pacing-induced decrement in the AP, relative AV node refractoriness upon pacing cessation due retrograde concealed penetration into the AV node following antidromic capture of the His bundle, and relative AV node refractoriness due to acceleration of the atrial rate following entrainment from the ventricle.^1,22^ Although correcting for delay in the AV node (corrected PPI) is helpful for short-R–P tachcyardias,^23^ it is less so for long-R–P tachycardias because it fails to account for the delay in the AP. (Of note, the corrected PPI can even be paradoxically longer than the uncorrected PPI if sufficient retrograde delay in the AP causes shortening of the first return AH interval relative to the tachycardia.) Therefore, the PPI − TCL < 125 ms criterion is specific for macroreentry (atypical ORT) but PPI − TCL > 125 ms is not necessarily specific for atypical AVNRT.

### Other criteria

Although ∆HA/∆VA criteria are useful to differentiate among short-R–P tachycardias, their value for long-R–P tachycardias has not been systematically validated.^21,24,25^ Pacing-induced AP decrement can lead to large ∆HA/∆VA values and the potential misdiagnosis of atypical AVNRT. In particular, several theoretical caveats exist for NF APs.

During PJRT/NVRT/NFRT, the HA interval is a true interval representing sequential activation of the HPS/AP/atrium. However, during entrainment from the ventricle with antidromic capture of the His bundle, the HA interval is a pseudo-interval reflecting parallel activation of the His bundle (over the HPS) and atrium (over the AP). Therefore, the ∆HA (HA _(entrainment)_ − HA _(PJRT/NVRT/NFRT)_) < 0 ms. In contrast, during atypical AVNRT, the HA interval is a pseudo-interval representing simultaneous activation of the His bundle and atrium but a true interval during entrainment from the ventricle, respectively, due to sequential activation of the His bundle and atrium over the AV node. Therefore, ∆HA (HA _(entrainment)_ − HA _(atypical AVNRT)_) > 0 ms.

During PJRT/NVRT, the VA interval is a true interval representing sequential activation of the HPS and ventricle/AP/atrium. During entrainment from the ventricle, the VA interval is also a true sequential interval. Therefore, the ∆VA (VA _(entrainment)_ − VA _(PJRT/NVRT)_) < 85 ms. In contrast, during atypical AVNRT, the VA interval is a pseudo-interval representing simultaneous activation of the ventricle and atrium but a true interval during entrainment from the ventricle due to sequential activation of the ventricle and atrium over the AV node. Therefore, ∆VA (VA _(entrainment)_ − VA _(atypical AVNRT)_) > 85 ms. During NFRT, the VA interval is actually a pseudo-interval and equals the conduction time from the NF to the atrium (retrogradely) minus the conduction time from the NF to the HPS and ventricle (antegradely). Therefore, the VA interval could be very short (theoretically, < 70 ms) with a very proximal RB insertion but can be longer and can approximate a true sequential interval with a distal RB insertion. During the entrainment of NFRT, the VA interval is a true interval. Therefore, theoretically, the ∆VA (VA _(entrainment)_ − VA _(NFRT)_) < 85 ms for distal NF insertion but possibly is > 85 ms for proximal NF insertion.

## Ablation

The target ablation site depends upon the mechanism of tachycardia and, therefore, it is critical to establish the exact diagnosis (eg, upper and lower limbs of the circuit). Conventional SP ablation during sinus rhythm targeting the SP or NF/NV AP–SP can effectively treat atypical AVNRT and NFRT/NVRT. In some cases, the circuit uses a left atrionodal pathway that requires a left-sided approach.^9^ Activation mapping is an alternative method targeting the atrial exit site of the SP (nodal tachycardia) or atrial insertion site of the AP (PJRT).

## Conclusions

The AV node-dependent long-R–P tachycardias impose unique diagnostic challenges because of the slow, decremental AP properties and unusual insertion sites in both the SP and RB. Conventional SVT criteria for short-R–P tachycardias are not always applicable for long-R–P tachycardias. The delivery of His-refractory VPDs is the most useful technique for identifying the presence of an AP, but both atrial and ventricular pacing maneuvers are required to accurately delineate the upper and lower limbs of the circuit **(Table 1)**.

## Figures and Tables

**Table 1:** Diagnostic Criteria of the Three AV Node-dependent AP-associated Long-R–P Tachycardias

	PJRT	NFRT	Atypical AVNRT + Bystander NF/NV AP
Upper limb (∆AH)	< 20 ms	> 40 ms	> 40 ms
		AH _(SVT)_ < AH _(NSR)_	AH _(SVT)_ < AH _(NSR)_
Lower limb (PPI − TCL)	< 125 ms	< 125 ms	> 125 ms

AH: atrio–His; AP: accessory pathway; AVNRT: atrioventricular nodal reentrant tachycardia; PJRT: permanent form of junctional reciprocating tachycardia; NF: nodofasciular; NFRT: nodofascicular reciprocating tachycardia; NV: nodoventricular; PPI: postpacing interval; TCL: tachycardia cycle length.
